# Performance Evaluation of Diagnostic Methods for *Schistosoma mansoni* Detection in Amhara Region, Northwest Ethiopia

**DOI:** 10.1155/2020/5312512

**Published:** 2020-10-18

**Authors:** Abebe Fenta, Tadesse Hailu, Megbaru Alemu, Arancha Amor

**Affiliations:** ^1^Health Science College, Debremarkos University, Debre Markos, Ethiopia P.O. Box 269; ^2^College of Medicine and Health Sciences, Bahir Dar University, Bahir Dar, Ethiopia P.O. Box 79; ^3^Mundo Sano Foundations, Institute of Health Carlos III, Madrid, Spain

## Abstract

**Background:**

*Schistosoma mansoni* is a parasitic worm that infects humans throughout the world. It is more prevalent in sub-Saharan Africa including Ethiopia. Proper detection of *Schistosoma mansoni* using sensitive diagnostic methods is crucial in the prevention and control era. Since direct wet mount microscopy with its low sensitivity has been used as a diagnostic technique in Ethiopia, searching alternative diagnostic methods which have better sensitivity should be a priority agenda.

**Objective:**

This study is aimed at evaluating the performance of diagnostic methods for *Schistosoma mansoni* in Amhara region.

**Methods:**

A cross-sectional study was conducted among 520 school children from October to December 2019 in Amhara region. The study participants were selected by a systematic random sampling technique. Stool samples were collected from each participant and processed via formol-ether concentration, Kato-Katz, and spontaneous tube sedimentation techniques. The data was entered into EpiData version 3.1, and analysis was done using SPSS version 20. The sensitivity, specificity, positive predictive value, and negative predictive value were calculated against the combined result as “Gold” standard. A kappa value was computed to measure the strength of agreement of the diagnostic methods.

**Results:**

The overall prevalence of *Schistosoma mansoni* was 20.2% using a combination of three methods. The prevalence of 8.3%, 12.9%, and16.3%, respectively, was recorded by using formol-ether concentration, Kato-Katz, and spontaneous tube sedimentation. The spontaneous tube sedimentation method (81.0%) had better sensitivity compared to Kato-Katz (63.8%) and formol-ether concentration (41.0%) methods in *Schistosoma mansoni* detection.

**Conclusion:**

The spontaneous tube sedimentation technique is more sensitive and has better detection rate to *Schistosoma mansoni* infection followed by the Kato-Katz technique. Therefore, updating the current diagnostic methods for *Schistosoma mansoni* could be a priority agenda to take action in schistosomiasis prevention and control.

## 1. Introduction

*Schistosoma mansoni* infection is one of the most important public health infections among the genus *Schistosoma* [1]. The global burden of schistosomiasis is estimated at 3.3 million disability-adjusted life years (DALYs) [2]. In Ethiopia, the number of people living in *Schistosoma* endemic areas is estimated at 37.3 million [3, 4]. The prevalence of *S. mansoni* infection within the Amhara region was 6.9% between 2011 and 2015 years [5].

*Schistosoma* parasites complete its life cycle in two different hosts, namely, humans and freshwater snails [6]. The human host acquires the infection via the active penetration of the skin by cercariae upon contact with fresh water infested with cercaria [7, 8]. Once *Schistosoma* enters the human body system, it infects chronically and results in chronic malnutrition and anaemia, especially in school-age children [9].

Infections of *S. mansoni* can be diagnosed by several diagnostic techniques including direct wet mount microscopy, Kato-Katz (KK), spontaneous tube sedimentation (STS), formol-ether concentration (FEC), and immunodiagnostic and molecular techniques. However, these methods vary in sensitivity, cost, simplicity, and applicability [10, 11]. The KK technique is recommended by the World Health Organization (WHO) as a means of detection method to *S. mansoni* infections [12, 13].

To make an effective diagnosis, diagnostic methods must be accurate, simple, and affordable for the whole diagnostic facilities and provide results within a short period in the clinical services [14]. In addition to patient management, higher sensitive diagnostic methods play an important role in the assessment of treatment efficacy [15]. Though molecular techniques have better performance than direct visualization techniques, its use is still limited in sub-Saharan African countries due to scarcity of resources. Likewise, low-income countries such as Ethiopia used low-sensitive diagnostic methods like the direct wet mount microscopic technique which leads to underdiagnose *S. mansoni* infection and might mislead the physicians [16–19]. This makes the current schistosomiasis prevention control strategies difficult and enforces us to find better sensitive diagnostic methods. To tackle this diagnostic challenge, updating better sensitive and cost-effective diagnostic methods as a routine diagnostic method is ideal in Ethiopia. Therefore, this study is aimed at evaluating the performance of FEC, KK, and STS for *S. mansoni* detection against the combined result (FEC, KK, and STS) as a “Gold” standard method.

## 2. Method

### 2.1. Study Design, Period, and Area

A cross-sectional study was conducted from October to December, 2019, in Amhara region. The Amhara region consists of 10 zones and 157 districts and is divided into three major ecological zones: the highlands, the midlands, and the lowlands. The annual mean temperature is between 15°C and 21°C. The mean annual rainfall is also 1,165 mm. According to the economic and finance office of the Amhara region 2017 report, the total population of children (5-14 years) in the region is 5,996,074 [20].

### 2.2. Inclusion and Exclusion Criteria

Students who fall in the age range of 6-14 years, gave consent, and volunteered to participate were included, whereas students who took antihelminthic drugs for the last 2 months prior to or during data collection time were excluded.

### 2.3. Sampling Techniques and Sample Size

A total of six districts and 11 primary schools were selected by simple random sampling technique, and school children in each school were also selected by systematic random sampling technique by using class roster as a sampling frame. The sample size in each school was proportionally allocated by considering the total number of students in each school. A total of 520 school children were included.

### 2.4. Data Collection and Processing

The study participants were informed about the purpose of the study. Approximately, a four-gram fresh stool sample was collected with a 25 millilitre stool cup from each study participant and transported to the nearby health institution within an hour. The fresh stool samples were processed with FEC, KK, and STS to detect *S. mansoni*. The intensity of *S. mansoni* infection (epg) was determined by the KK method only due to its feasibility.

In the modified Richie's method, approximately half a gram of fresh stool sample was added in the sample collecting tube containing two and half millilitres of formalin and one millilitre of ethyl acetate. It was mixed well and centrifuged at 1500 revolutions for three minutes. Finally, the supernatant was discarded and the sediment was mixed and put on a microscope slide to detect the ova of S. mansoni using a microscope [21].

In the KK technique, a stool sample was pressed through a mesh screen to remove large particles. About 41.7 milligrams of sieved stool was transferred to the template which was put on a slide until the template hole is filled. Then, the template was removed and the stool sample was covered with cellophane (previously immersed with glycerol-malachite green) and pressed with a new slide. In the KK thick smears, *S. mansoni* ova were examined and the infection intensity was estimated based on the eggs per gram (EPG) of stool. The intensity of *S. mansoni* is categorized as light (1–99 EPG), moderate (100–399 EPG), and heavy (>400 EPG) based on the WHO criteria [13].

In the STS technique, approximately three grams of fresh stool sample was weighed and homogenized in 10 ml of normal saline solution. The mixture was filtered through surgical gauze into a 50 ml plastic tube which was then filled with more saline solution up to 50 millilitres, plugged, and shaken vigorously. The tube was left to stand for 45 minutes, and then, the supernatant was discarded. A sample was taken from the bottom and put on a microscopic slide and seen for the ova of *S. mansoni* by a microscope [11].

### 2.5. Performance Evaluation

The detection rate and performance of FEC, STS, and KK methods to *S. mansoni* were checked by taking the combined result as a “Gold” standard [22]. Since there is no reference method for *S. mansoni*, the sensitivity, specificity, negative predictive values, and positive predictive values were calculated for each of the three methods considering the combined results from the individual methods (e.g., any positive value from the three methods was regarded as positive) as the diagnostic “Gold” standard. The diagnostic agreements of diagnostic methods were evaluated by the Kappa value. Kappa result was interpreted as follows: values ≤ 0 as indicating no agreement and 0.01–0.20 as none slight, 0.21–0.40 as fair, 0.41– 0.60 as moderate, 0.61–0.80 as substantial, and 0.81–1.00 as perfect agreement [23].

### 2.6. Data Quality Control

Training was given for laboratory personnel about the study, data collection, and detection. The quality of reagents and instruments was checked. The stool samples were also checked for their serial number and quantity. To eliminate observer bias, each stool sample was examined immediately by two laboratory personnel. To maintain the reliability of the study findings, 15% of the KK slides were randomly selected and reexamined by a third laboratory personnel who was blind for the first stool examination. The principal investigator also checked the discordant results and puts the final result.

### 2.7. Data Analysis

The data was entered in EpiData version 3.1 and analysed using SPSS version 20.0 statistical software for descriptive statistics. The sensitivity (SN), specificity (SP), negative predictive value (NPV), and positive predictive value (PPV) for each diagnostic method in *S. mansoni* detection were calculated against the combined results as a “Gold” standard method. Kappa values were estimated at 95% CI to determine the strength of agreement of the diagnostic methods. *p* value < 0.05 was considered statistically significant.

### 2.8. Ethical Consideration

Ethical clearance was obtained from the College of Medicine and Health Science Ethical Review Committee, Bahir Dar University. Permission letter was obtained from Amhara Regional Health Bureau. Supporting letters were also secured from Amhara Regional Education Bureau, Zonal, and District Education Offices. Written informed consent was secured from parents/guardians of each study participant, and assent was also obtained from each study participant. Study participants infected with intestinal parasites were referred to the nearby health institution for treatment.

## 3. Results

### 3.1. Sociodemographic Characteristics of the Study Participants

A total of five hundred twenty (*n* = 520) students were enrolled in this study. The mean age was 10.14 years ranged from 6 to 14 years with a standard deviation of 1.66 years. The male participants accounted for 266 (51.2%), and four hundred ninety-seven (95.6%) participants were rural dwellers.

### 3.2. Prevalence of *Schistosoma mansoni*

The overall prevalence rate of *S. mansoni* was 20.2% with a combined method. The detection rate of 16.3%, 12.9%, and 8.3% to *S. mansoni* infection was obtained using the STS, KK, and FEC techniques, respectively (Table 1).

**Figure 1:**
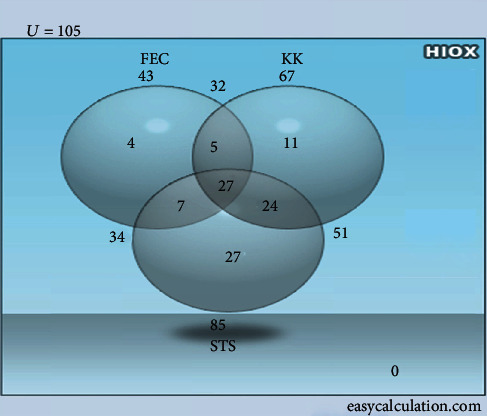
Performance of FEC, KK, and STS techniques in the detection of S. mansoni infection.

The STS method detected 27 samples that were negative by the KK and the FEC methods. It also detected 4 and 11 false negative samples that were positive by the FEC method and KK method, respectively (Figure 1). On the other hand, the KK method detected 11 samples that were negative by the FEC method and the STS method. The STS, KK, and FEC methods were similar in the detection of 27 infected individuals (Figure 1).

The intensity of S*. mansoni* infection was calculated after converting the number of eggs counted in 0.0417 g of stool by KK thick smear into EPG. About 49 (72.3%) *S. mansoni* infections were light. The mean EPG for *S. mansoni* infection observed was 69.15, ranging from 24 to 792 (Table 2).

### 3.3. Detection and Performance Evaluation of Diagnostic Methods for *S. mansoni*

The detection rate of the combined method to *S. mansoni* was 2.44, 1.57, and 1.24 times more sensitive than the FEC, KK, and STS methods, respectively. The STS method was 1.26 and 1.98 times more sensitive in *S. mansoni* detection than the KK and FEC methods, respectively (Table 3).

The detection rate by a combination of STS and KK (19.4%) to *S. mansoni* was better compared to the other two combined techniques (KK + FEC and that of STS + FEC) (Table 4). The STS technique had more sensitivity (81.0%) and better NPV (95.4%) than the respective KK sensitivity (63.8%) and NPV (91.6%) and FEC sensitivity (41.0%) and NPV (87.0%) in the identification of *S. mansoni*. The sensitivity of KK technique (63.8%) was higher than FEC technique sensitivity (41%) for the diagnosis of *S. mansoni* infection. However, specificity and positive predictive value of detecting the *S. mansoni* parasites were similar (100%) in all the three techniques (Table 3).

The agreement of STS technique with the combined results was perfect in detecting *S. mansoni* (*κ* = 0.872). The KK method agreed substantially in *S. mansoni* (*κ* = 0.738) identification with the combined techniques. The FEC technique agreed moderately in *S. mansoni* (*κ* = 0.525) detection with gold standard (Table 5).

## 4. Discussion

Highly sensitive diagnostic methods are necessary for the diagnosis of helminthic infections in the routine clinical service to monitor treatment outcomes and for intervention purposes [24]. In the present study, the prevalence of *S. mansoni* was 20.2% which is comparable with the previous studies done in Ethiopia (20.2%) [25] and Kenya (21%) [26], but higher than previous study results (14.4%) in Brazil [27], (12.1%) in Nigeria [28], (0.75%) in Homesha district, Western Ethiopia [29], and (10.3%) in Jawi district, Northwest Ethiopia [30]. On the other hand, higher prevalence of *S. mansoni* was recorded in Lake Hawassa, Southern Ethiopia (31%), compared to the present study [31]. The differences could be due to the difference in geographical location, snail distribution, local endemicity of the parasite, and laboratory techniques used.

The intensity of *S. mansoni* infection in the present study shows light (73.1%), moderate (23.9%), and heavy (3.0%) among the total *S. mansoni* positive school children which is comparable with a study done in Azezo (light 67.8%, moderate 19.8%, and heavy 3.1%) [32], but it is quite different from Sanja, Northwest Ethiopia (18.4% light, 47% moderate, and 18.7% heavy) [8]; this might be due to the variation of infection rate.

The STS technique is the simplest, fastest method to perform, requires less equipment, and detects many species [11]. In the present study, the STS method was 1.26 and 1.98 times more sensitive in *S. mansoni* detection than the KK and FEC methods, respectively. Although there are insufficient data which have been conducted on the detection rate of this method before, the result obtained in our study supporting the STS technique can be considered an alternative diagnostic method for S*. mansoni* infections.

In the present study, *S. mansoni* was found in 8.3% of the students using the FEC technique and 12.9% by the KK method. The difference in their diagnostic sensitivity is statistically significant (*p* = 0.001). This result agrees with previous studies done in Peusangan Bireuen [33] and Ethiopia [34, 35] which stated that the FEC method was less sensitive than the KK method. However, the result of the present study is in contrary with the study done in Côte d'Ivoire [36] and Tanzania [24] which showed that the FEC method is more sensitive than the KK method. There are also similar reports in Ethiopia which stated that FEC has higher sensitivity than KK to detect *S. mansoni*[25, 37]. The differences might be due to the type of stool sample (being formed or diarrhoea), number of samples collected and slides prepared for diagnosis, interpersonal skill variations, and technical errors of the diagnostic methods used.

Taking the combined results of three techniques as a “Gold” standard, the STS technique (81%) had higher sensitivity than the KK (63.8%) and FEC (41%) techniques in the detection of *S. mansoni* infection. However, the lack of previous similar studies made difficulty in making rigorous discussion on this finding. On the other hand, the sensitivity of KK (63.8%) and FEC (41%) was recorded in *S. mansoni* detection. This showed that the KK method was 1.55 times more sensitive than the FEC method in the diagnosis of *S. mansoni* infection in the current study, as previously confirmed by Glinz et al., which is 2 times more sensitive than the KK method in Côte d'Ivoire [36].

The agreement of the STS technique with the combined results was perfect in detecting *S. mansoni* infections (*κ* = 0.872). On the other hand, the agreement of KK (*κ* = 0.738) with the gold standard was substantial for the detection of *S. mansoni*. There was difficulty in making rigorous discussion on these findings due to lack of previous reports. The agreement of the FEC technique with the “Gold” standard to detect *S. mansoni* was moderate (*κ* = 0.525) which is comparable with the previous study done in Gondar town (*κ* = 0.58) [34].

## 5. Conclusion

The present study revealed that the STS method has a better detection rate and performance compared to the FEC and KK methods in *S. mansoni* detection. In addition, the KK method showed better performance compared to the FEC technique to detect *S. mansoni*. Therefore, updating the current diagnostic methods for *Schistosoma mansoni* could be a priority agenda to take action in the schistosomiasis prevention and control. Furthermore, implementing the STS technique as a routine laboratory diagnosis of stool aids to increase the detection rates of *S. mansoni* infection in endemic areas.

## Figures and Tables

**Table 1:** Prevalence of S. mansoni using combined, FEC, KK, and STS techniques among school children in ANRS, from October to December, 2019 (*n* = 520).

*S. mansoni* diagnostic technique	Prevalence detected by each method		
	*N*	%	95% CI
Combined	105	20.2	16.7-23.7
FEC	43	8.3	5.9-10.6
KK	67	12.9	10-15.8
STS	85	16.3	13.2-19.5

*N* = number of positives; CI = confidence interval.

**Table 2:** Intensity of *S. mansoni* infection using KK technique among school children in ANRS, from October to December, 2019 (*n* = 520).

Infection intensity	*S. mansoni*	
	*N*	%
Light	49	73.1
Moderate	16	23.9
Heavy	2	3.0
Total positive	67	12.9

*N* = number of positives; % = percentage.

**Table 3:** Detection rate and performance of FEC, KK, and STS techniques to diagnose *S. mansoni* against the gold standard among school children in ANRS, from October to December, 2019 (*n* = 520).

Method	Result	“Gold” standard		Sensitivity	Specificity	NPV	PPV
		Pos	Neg	% (95% CI)	% (95% CI)	% (95% CI)	% (95% CI)
FEC	Pos	43	0	41.0 (32-50.5)	100 (99.1-100)	87.0 (83.7-89.7)	100 (91.8-100)
	Neg	62	415				
KK	Pos	67	0	63.8 (54.3-72.4)	100 (99.1-100)	91.6 (88.7-93.8)	100 (94.6-100)
	Neg	38	415				
STS	Pos	85	0	81.0 (72.4-87.3)	100 (99.1-100)	95.4 (93.0-97.0)	100 (95.7-100)
	Neg	20	415				

CI: confidence interval; PPV: positive predictive value; NPV: negative predictive value.

**Table 4:** Prevalence of *S. mansoni* parasites diagnosed as using FEC, KK, and STS individually and their combinations among school children in ANRS, from October to December, 2019 (*n* = 520).

Method	*S. mansoni*	
	Pos (*N* (%))	% (95% CI)
FEC	43 (8.3)	5.9-10.6
KK	67 (12.9)	10-15.8
STS	85 (16.3)	13.2-19.5
FEC + KK	78 (15)	12.2-18.3
FEC + STS	94 (18.1)	15-21.6
KK + STS	101 (19.4)	16.2-23.1
FEC + KK + STS	105 (20.2)	17.0-25.1

Pos = positive; Neg = negative; *N* = number.

**Table 5:** Test agreement of FEC, KK, and STS techniques to detect *S. mansoni* against the gold standard among school children in ANRS, from October to December, 2019 (*n* = 520).

Method	Result	“Gold” standard		Kappa-value (*p* value)	95% CI of kappa
		Pos (*N*)	Neg (*N*)		
FEC	Pos	43	0	0.525 (0.001)	0.428-0.623
	Neg	62	415		
KK	Pos	67	0	0.738 (0.001)	0.660-0.815
	Neg	38	415		
STS	Pos	85	0	0.872 (0.001)	0.817-0.926
	Neg	20	415		

Pos = positive; Neg = negative; *N* = number; SM = *S*.*mansoni*; CI = confidence interval.

## Data Availability

Minimal data could be accessed upon request.

## Abbreviations

DALYs: Disability-adjusted life years
EPG: Eggs per one gram
FEC: Formol-ether concentration
KK: Kato-Katz
NPV: Negative predictive value
PPV: Positive predictive value
STS: Spontaneous tube sedimentation
WHO: World Health Organization.
